# Kanamycin and Cisplatin Ototoxicity: Differences in Patterns of Oxidative Stress, Antioxidant Enzyme Expression and Hair Cell Loss in the Cochlea

**DOI:** 10.3390/antiox11091759

**Published:** 2022-09-06

**Authors:** Alejandro Gibaja, Juan C. Alvarado, Verena Scheper, Liliana Carles, José M. Juiz

**Affiliations:** 1 Instituto de Investigación en Discapacidades Neurológicas (IDINE), School of Medicine, Universidad de Castilla-La Mancha (UCLM), Campus in Albacete, 02008 Albacete, Spain; 2 Department of Otorhinolaryngology, Head and Neck Surgery, Hannover Medical School, 30625 Hannover, Germany; 3 Cluster of Excellence “Hearing4all” of the German Research Foundation, DFG, MHH, 30625 Hannover, Germany; 4 Department of Otolaryngology, University Hospital “Doce de Octubre”, 28041 Madrid, Spain; 5 IDINE/Med School, UCLM-Campus in Albacete, C/Almansa 14, 02008 Albacete, Spain

**Keywords:** deafness, cochlear toxicity, antibiotic toxicity, antioxidation, auditory receptor

## Abstract

Kanamycin and cisplatin are ototoxic drugs. The mechanisms are incompletely known. With subcutaneous kanamycin (400 mg/kg, 15 days), auditory threshold shifts were detected at days 12–13 at 16 and 32 kHz, extending to 8 and 4 kHz at days 14–15. The outer hair cell (OHC) loss was concentrated past day 12. The maximum cochlear length showing apoptotic cells, tested with TUNEL, was at day 13. At day 15, 1/5 of the apical cochlea contained preserved OHCs. 3-nitrotyrosine (3-NT) immunolabeling, showing oxidative stress, was found in surviving OHCs and in basal and middle portions of the stria vascularis (SV). The antioxidant Gpx1 gene expression was decreased. The immunocytochemistry showed diminished Gpx1 in OHCs. With intraperitoneal cisplatin (16 mg/kg, single injection), no evoked auditory activity was recorded at the end of treatment, at 72 h. The basal third of the cochlea lacked OHCs. Apoptosis occupied the adjacent 1/3, and the apical third contained preserved OHCs. 3-NT immunolabeling was extensive in OHCs and the SV. Gpx1 and Sod1 gene expression was downregulated. Gpx1 immunostaining diminished in middle and basal SV. More OHCs survived cisplatin than kanamycin towards the apex, despite undetectable evoked activity. Differential regulation of antioxidant enzyme levels suggests differences in the antioxidant response for both drugs.

## 1. Introduction

Around 200 drugs may cause hearing loss [1,2]. Among them, two groups with high ototoxic potential, aminoglycoside antibiotics and cisplatin and its derivates, are widely used in different therapeutical applications [2].

Aminoglycoside antibiotics are the first choice in the treatment of many resistant infections caused by Gram^-^ bacteria. They are relatively inexpensive to produce, being extensively used in world regions with limited resources [3,4]. Besides their bactericidal power, they are also used to treat genetic diseases caused by abnormal stop codons, such as Duchenne muscular dystrophy, due to their ability to shift protein reading frames in the ribosome [4,5]. Despite their recognized ototoxicity [2,6], the benefits of aminoglycosides encourage protection from hearing loss [6], instead of abandonment, as therapeutic agents.

Coordination complexes of platinum, particularly cisplatin, are extensively used as anti-neoplastic drugs. Despite advances in targeted cancer therapies, cisplatin, alone or in combined chemotherapy cycles, is still an essential part of oncotherapy. This includes treatment of solid tumors, notably testicular, ovarian and neck cancer as well as pediatric malignancies such as medulloblastoma, osteosarcoma or germ cell tumors [7,8].

For both types of drugs, much knowledge has accumulated on mechanisms of ototoxicity, although they are not yet fully worked out [6,9,10]. Clearly, pharmacological and ototoxicity mechanisms are linked. In the case of aminoglycosides, bactericidal effects are mediated by interference with protein synthesis through binding to one of the subunits of the prokaryotic cell ribosome. One key to ototoxicity is that in eukaryotic cells, aminoglycosides interact with mitochondrial rRNA in ribosome subunits, which is evolutionarily related with its prokaryotic counterparts. Mitochondrial protein synthesis is then altered [11,12], and the activity of enzymes in the electron transport chain involved in oxidative phosphorylation is compromised, as shown in the cochlea [10,13], leading to faulty energy metabolism. Oxidative imbalance leads to excess accumulation of incompletely reduced, and thus avidly reactive, oxygen-related radicals or reactive oxygen species (ROS) [14,15,16,17]. Among other things, they readily combine with nitrogenated compounds, giving rise to nitrogen-related radicals or reactive nitrogen species (RNS). Therefore, ROS and RNS are free radicals that contribute fundamentally to unchecked oxidative stress, which finally overrides cellular “antioxidant defenses” [4,16,17,18]. Thus, oxidative stress self-perpetuates, irreversibly altering gene expression, as DNA and RNA are prone to oxidative damage, and the molecular structure, metabolism and interactions of proteins and complex lipids, which disorganizes cell membranes and membrane compartments [16,17,18]. Altogether, this results in the activation of pathways leading to cell death, notably, although not exclusively, apoptosis, as shown in the cochlea [19]. In such an oxidative imbalance context, the elevated metabolic demands of electromechanical transduction in the auditory organ may contribute to ototoxicity by more easily tipping out of balance the scale of oxidative stress [12].

Regarding cisplatin, its antitumoral mechanisms involve the ability to crosslink with DNA, which blocks DNA replication and repair. Mitochondrial DNA, which lacks histones, easily forms such DNA adducts with cisplatin. The mitochondrial function is therefore compromised in tumoral cells, resulting in excess accumulation of ROS/RNS, leading to oxidative stress and activation of pathways leading to cell death [3,7]. Other mechanisms such as direct inactivation of antioxidant enzymes, may also contribute importantly to cisplatin antitumoral cytotoxicity [20]. However, by virtue of its mechanisms of action, antitumoral cisplatin cytotoxicity is accompanied by unwanted cytotoxicity on normal cells. Ototoxicity is a major secondary effect of cisplatin treatment, which may lead to irreversible deafness in a very significant number of patients [4,21]. Damaging cisplatin-DNA adducts form in cochlear sensory and supporting cells [21,22]. The disruption of mitochondrial integrity and buildup of ROS/RNS with ensuing oxidative stress are also central to cisplatin ototoxicity [4,8,10]. Such as in the case of aminoglycosides, the delicate metabolic energy balance in the auditory receptor also contributes to cisplatin ototoxicity. In addition, specific factors, such as the binding of cisplatin to the NADPH-oxidase NOX3, which is almost exclusively expressed in the inner ear, make the auditory receptor particularly prone to cisplatin toxicity [10,23].

In sum, much evidence supports that unchecked oxidative stress is at the core of irreversible hearing loss caused by aminoglycosides and cisplatin and other ototoxic drugs, as well as acoustic trauma and auditory aging [12]. Physiologically, oxidative stress is kept within narrow margins by non-enzymatic and enzymatic mechanisms. “Antioxidant” non-enzymatic mechanisms include free radical scavengers, which are compounds with high potential for redox reactions with free radicals, preventing them from further intracellular redox interactions, deleterious for the function of essential macromolecules. Such “antioxidants” include glutathione and related compounds as well as some vitamins [17]. Enzymatic mechanisms include enzyme systems that catalyze redox reactions that regenerate free radical scavengers or inactivate free radicals such as ROS/RNS. Among them, Glutathione peroxidase 1 (Gpx1), Catalase (Cat) and the superoxide dismutase (SOD, particularly SOD1) have a central role in controlling oxidative stress. SOD catalyzes the conversion of O_2-._to H_2_O_2,_ whereas Cat catalyzes the conversion of H_2_O_2_ in H_2_O and O_2._ Gpx1 also catalyzes the reduction of H_2_O_2_ and other peroxides, as well as the “regeneration” of reduced glutathione to its oxidized form after H_2_O_2_ conversion [17]. There is evidence showing involvement of these enzymes in ototoxicity. For instance, changes in GPx1, catalase or SOD1 expression or activities, linked to aminoglycoside or cisplatin hearing loss, have been reported [10,24,25]. However, it is not known whether the expression and cellular distribution of these canonical antioxidant enzymes are identically affected regardless of the nature of the ototoxic agent, or whether there are differences. This is due in part to limited availability of experimental studies in animal models “in vivo,” in which the sequence of ototoxic damage in relation to the expression of antioxidant enzymes is compared directly side by side, for aminoglycosides and cisplatin. The question is relevant because differences or similarities may have implications for the mechanistic understanding of ototoxic damage, which may potentially translate into improved therapeutic strategies.

Therefore, we have compared in the Wistar rat model the ototoxicity of the aminoglycoside antibiotic kanamycin with that of cisplatin in relation to the generation of oxidative stress and the expression and tissue localization of the antioxidant enzymes Gpx1, Cat and SOD1. For this, we have used a combination of RT-qPCR, quantitative histology and immunocytochemistry with antibody markers of oxidative stress and antioxidant enzymes, along with acoustically evoked auditory brainstem response recordings (ABRs).

## 2. Materials and Methods

### 2.1. Experimental Animals

Male Wistar rats, aged from 11 to 13 weeks old at the beginning of the experiments, were used. Animals were provided by Charles River Laboratories (Barcelona, Spain) and were housed in the Animal House facility of the University of Castilla-La Mancha-UCLM in Albacete (Spain) under controlled temperature (22–23 °C) and humidity (60% ± 5%) in a 12-h light/dark cycle with ad libitum access to food and water. The handling and care of animals followed current national (Spain R.D. 53/2013; Law 32/2007) and EU (Directive 2010/63/EU) regulations on the protection and care of animals used for scientific purposes. Procedures were validated and supervised by the institutional Ethics Committee for Animal Experimentation and were authorized under registry PR-2016-04-11.

### 2.2. Induction of Ototoxicity by Kanamycin and Cisplatin

Kanamycin (Sigma-Aldrich, St. Louis, MO, USA, No. Cat. K4000) in 0.9% saline was injected daily subcutaneously at 400 mg/kg, from 3 to 15 days, depending on the survival time to which animals were randomly assigned (see below). Animals in the control group received saline vehicle injections.

Rats were randomly distributed in the following groups: Kanamycin treatment, 3 days (D3; *n* = 12), 11 days (D11; *n* = 12), 12 days (D12; *n* = 3), 13 days (D13; *n* = 3), 14 days (D14; *n* = 3) and 15 days (D15; *n* = 11). The saline vehicle-injected control group was 15 days (*n* = 10). Prior to the first administration of the drug, auditory brainstem response (ABR) recordings were carried out to confirm normal baseline from controls, as previously published [26]. Immediately before euthanasia, a second ABR recording was performed to analyze changes in hearing thresholds after the corresponding days of treatment. After recording ABRs at the specified treatment days, eight cochleae from 8 animals in the D3, D11 and D15 survival groups were processed for OHC counts and apoptosis detection with the TUNEL method, whereas the other eight cochleae were processed for immunocytochemical detection of 3-NT and Gpx1 (see below), as described below. In addition, to establish the timeline of OHC loss, the cochleae of the 3 animals from the D12, D13 and D14 survival groups were processed for OHC counts and TUNEL staining (see below). Controls were cochleae from 6 animals in the saline vehicle-injected group.

The cochleae from the remaining 4 animals in the D3, D11 and D15 survival groups along with 4 animals in the control group were used for the gene expression analysis of antioxidant enzymes using RT-qPCR, as described in the corresponding section below.

Cisplatin (Sigma-Aldrich, Cat. N. P4394) was administered at 16 mg/kg in 0.9% saline through a single intraperitoneal injection [27]. Animals were randomly distributed in two survival groups: cisplatin 24 h (24 h; *n* = 9) and cisplatin 72 h (72 h; *n* = 9). The control group was injected intraperitoneally with saline vehicle and allowed to survive 72 h (*n* = 10). Prior to cisplatin injection, ABR recordings were performed to confirm hearing sensitivity within the Wistar rat normal range [26]. Prior to sacrifice (i.e., 24 or 72 h after administration of cisplatin) a second ABR recording was performed to test the effects of cisplatin on hearing function. Five cochleae from 5 animals in each survival group (24 and 72 h) were used for OHC cell counts and TUNEL staining. The other five cochleae from the animals in each survival group were used for 3-NT and Gpx1 immunocytochemistry. Five saline-injected animals were used as controls. The cochleae of the remaining four animals in each survival group and from five control animals were used for RT-qPCR. The final sample number was pooled from cisplatin injection protocols with mortality rates from 32% to 44%, within the range or lower than previously reported with similar experimental protocols in rats [24].

### 2.3. Auditory Brainstem Response Recordings (ABRs)

ABR recordings were carried out in a sound-proof booth (Incotron Eymasa S.L., Barcelona, Spain) inside a sound-attenuation room, as previously described [26,28]. Animals were anesthetized with isoflurane at a flow rate from 1 L/min O_2_ at 4% for induction and 1.5–2% for maintenance. During the recordings, body temperature was regulated at 37.5 °C with a non-electrical thermal pad. Temperature was monitored and controlled with a rectal probe. The subdermal electrodes (Rochester Electro-Medical, Tampa, FL, USA) were placed at the cranial vertex (non-inverted) and on the right (inverted) and left (ground) mastoid apophysis. Sound stimulation and recordings were carried out using a BioSig System III (Tucker-Davis Technologies, Alachua, FL, USA).

The stimuli were generated digitally with SigGenRP software (Tucker-Davis Technologies) and an RX6 Piranha Multifunction Processor hardware (Tucker-Davis Technologies). Stimuli were tone bursts (5 ms rise/fall without a plateau, 20 times/second) at seven different frequencies (0.5, 1, 2, 4, 8, 16 and 32 kHz), which were applied into the right external ear canal using an ED1 electrostatic speaker controller (Tucker-Davis Technologies) through an EC-1 electrostatic speaker (Tucker-Davis Technologies). The stimuli were calibrated prior to experiments with SigCal software (Tucker-Davis Technologies) and an ER-10B+ low-noise microphone (Etymotic Research Inc., Elk Grove, IL, USA).

Auditory thresholds were obtained for each tested frequency. To determine threshold level, the evoked responses were recorded in 5 dB steps descending from a maximum stimulus intensity of 80 dB SPL. The background activity was measured before the stimulus onset. The auditory threshold was defined as the stimulus intensity that evoked waveforms with a peak-to-peak voltage greater than 2 standard deviations (SD) of the background activity [26,28]. The maximum level of stimulus intensity was established at 80 dB as a safe limit for noise overstimulation [28,29]. Following treatments, if no evoked responses were obtained at 80 dB, the auditory thresholds were recorded at that value for statistical analysis [29].

The threshold shift was determined for each of the frequencies studied by subtracting the auditory thresholds at the different survival times after treatments from auditory thresholds obtained prior to the initiation of treatment.

### 2.4. Real Time Quantitative PCR

Animals intended for the analysis of gene expression in the cochlea received an overdose of sodium pentobarbital (700 mg/kg, intraperitoneal). The cochleae were extracted, quickly frozen in liquid nitrogen, and stored at −80 °C until they were used for real time quantitative PCR (RT-qPCR).

#### 2.4.1. RNA Extraction and cDNA Synthesis for RT-qPCR

First, 1 mL of cold TRIzol reagent (Thermo-Fisher Scientific, Waltham, MA, USA) was added to each frozen cochlea, according to the manufacturer’s instructions. Then, they were then quickly homogenized using a Polytron PT 2100 homogenizer (Kinematica-Fisher Scientific, Malters, Switzerland) for 30–40 s. The pre-cleaned Polytron rotor was treated with RNaseZap (Sigma-Aldrich, St. Louis, MO, USA) and cooled with dry ice for approximately 1 min. After 5 min incubation at room temperature, 0.2 mL chloroform was added, followed by re-incubation for 3 min at room temperature. Samples were then centrifuged for 15 min at 12,000× *g* at 4 °C. After this, the upper aqueous phase was transferred to a new tube to which 0.5 mL of isopropanol was added. Incubation proceeded for 10 min at room temperature. Samples were then centrifuged at 12,000× *g* for 10 min at 4 °C to precipitate RNA, the supernatant was removed, and the pellet was resuspended in 1 mL of 75% ethanol. After vortex-washing, the pellet was re-centrifuged at 7500× *g* for 5 min at 4 °C, the resulting supernatant was removed, and the pellet was air-dried for 5–10 min. Finally, the pellet was re-suspended in 20 mL of RNAse-free H_2_O and incubated at 55 °C for 10 min. The quantity and quality of RNA were tested by spectrophotometry (Nanodrop ND-1000, Thermo Scientific). All RNA samples showed appropriate A260/A280 ratios. The RNA was stored at −80 °C.

For cDNA synthesis, RevertAid First Strand kit (Thermo Fisher Scientific), was used, with 1 g of RNA with oligo–(dT)18 as a primer. Reactions were carried out on a DNA Engine^®^ Peltier thermocycler (Bio-Rad, Hercules, CA, USA) at a final volume of 20 mL. The reaction conditions were as follows: 65 °C for 5 min, 37 °C for 5 min, 42 °C for 1 h, 72 °C for 10 min and 4 °C until tube collection. The reaction product was diluted 1:10 in ultrapure water for qPCR use and remained at 4 °C for immediate use or at −20 °C for long-term storage. All qPCR experiments were conducted with the same batch of cDNAs. Controls without reverse transcription (–RT) were carried out to verify the absence of genomic DNA contamination.

#### 2.4.2. RT-qPCR

RT-qPCR was carried according to previously published methods [30]. A StepOnePlus Real-Time PCR System (Applied Biosystems, Waltham, MA, USA) was used, with 96-well plates and Fast SYBR Green Master Mix (Applied Biosystems) as the detection reagent. The reaction mixture in each well consisted of 2.8 mL of ultrapure water, 0.1 mL of each primer (final concentration of 100 nM), 5 mL of Fast SYBR Green Master Mix and 2 mL of 1:10 cDNA. The plate was centrifuged at 1200 rpm for 2 min. PCR amplification was performed under the following conditions: initial activation at 95 °C for 20 s, followed by 40 cycles of 95 °C for 6 s and 60 °C for 45 s. To confirm that the primers amplified a single specific PCR product, a melting curve was generated by an initial denaturation step at 95 °C for 20 s, followed by a gradual heating of 60 °C to 95 °C, in increments of 0.3 °C. All qPCR plates included controls without cDNA (NTC) that generated values of Cq > 35. The experiments were performed in triplicate.

Cq data were obtained using Step One v2.3 software (Applied Biosystems). The level of expression of a target gene was obtained following the ∆∆Cq method [31]. It was normalized to the average level of the reference gene, in our case *Gapdh* [30,32], obtaining the value of ∆Cq for each pair of genes in the samples (control and treatment). The ∆∆Cq of each gene was calculated according to:

∆∆Cq = ∆Cq (treatment group) − ∆Cq (control group)

Where the “treatment group” corresponds to each experimental treatment group: cisplatin 24 h and 72 h; kanamycin D3, D11 and D15. Relative expression was calculated according to:

Relative Expression = 2^∆∆Cq^.

qPCRs were performed using pairs of specific primers to amplify the transcription of genes related to antioxidant enzymes. Pairs of primers (Table 1) were designed using Primer3 Plus software (http://www.bioinformatics.nl/cgi-bin/primer3plus/primer3plus.cgi/, accessed date 4 July 2022) and were produced by Thermo Fisher Scientific. Specificities were tested using BLAST analysis (NIH, NCBI, USA). Using Ensembl databases, it was verified that primers covered at least two exons with a large intron in between to prevent false amplification resulting from genomic DNA contamination.

### 2.5. Cochlear Histology

Animals intended for histological analysis of the cochlea were anesthetized with sodium pentobarbital (Dolethal, Vetoquinol, Madrid, Spain; i.p. 700 mg/kg) after ABR recordings. Tissue fixation by transcardial perfusion was initiated with a vascular flush with 0.9% saline solution, followed by 4% paraformaldehyde fixative (PFA 4%) in 0.1 M phosphate buffer (PB, pH 7.3). After perfusion, the cochleae were dissected out from the temporal bones, after which they were washed three times in phosphate-buffered saline (PBS) for 5 min and decalcified in 50% RDO (Rapid Decalcifier Solution, Apex Engineering Products Corporation, IL, USA). The right cochleae, used for quantification of outer hair cells (OHCs) and apoptosis, were maintained in RDO for 1 h. The left cochleae, used for immunohistochemistry (Table 2) in 20 μm-thick cochlear sections, were maintained in RDO for 2 h. After decalcification, the cochleae were washed in PBS 3 times for 5 min.

#### 2.5.1. Cochlear Spiral Surface Preparations for Hair Cell Quantification and TUNEL Method for Detection of Apoptosis

The right cochleae were dissected under an Olympus SZX10 stereoscopic microscope (Olympus, Tokyo, Japan) with the help of 2 thin-tipped dissection tweezers and a surgical blade. First, a cut was made with the blade perpendicular to the modiolus, dividing the total length of the cochlea in two equal halves. For the apical half, the decalcified bone capsule was removed with the help of tweezers. Then, the lateral wall was excised and the modiolus and the tectorial membrane were removed. For the basal half, the procedure was similar, with special care when removing lateral wall structures from the bone in the most basal segment. Each dissected cochlear turn fragment (between three and four for the full cochlear length) with the organ of Corti exposed, was immersed in PBS in a single well of a multi-well plate (Sigma-Aldrich).

To visualize and count OHCs (see below), immunofluorescence for the detection of myosin VIIa was performed on the cochlear segments “in toto”. The TUNEL method for detection of apoptosis (*Terminal Deoxynucleotidyl Transferase dUTP Nick End L*abeling) was used simultaneously.

Cochlear turn fragments were first incubated in phosphate-buffered saline with 1% TritonX-100 and 5% bovine serum albumin (PBS-Tx100 1%-BSA 5%) for 1h. They were then incubated with an anti-myosin-VIIa primary antibody (Proteus BioSciences, Ramona, CA, USA) diluted 1:1000 in PBS-Tx100 0.2%-BSA 3%. The incubation proceeded overnight. The next day, the primary antibody solution was removed, and the cochlear turns were washed in PBS 3 × 5 min. This was followed by incubation in anti-rabbit Alexa Fluor 594 secondary antibody (Thermo Fisher Scientific) diluted 1:200 in PBS-Tx100 0.2%-BSA 3% for 1 h at room temperature in darkness. After removing the secondary antibody, the cochlear turns were washed once in PBS-Tx100 0.2% for 5 min, and 3 times more in PBS for 5 min. After washing, TUNEL was carried out on the same samples, using the DeadEnd kit™ Fluorometric TUNEL System (Promega, Madison, WI, USA). After removing the PBS from the wells, 50 mL of “equilibration” buffer was added, and the cochlear turns were incubated for 10 min. After removing the “equilibration” buffer, 25 mL of reaction mix (*TdT reaction mix*: “equilibration buffer”, 22.5 mL, nucleotide mixture, 2.5 mL, rTdT enzyme, 0.5 L) were used to incubate for 50 min at 37 °C in the darkness and in a moist chamber. The reaction was stopped by adding 400 mL of SSC 2X (Stop*Reaction Buffer*) and incubating for 15 min. Finally, the cochlear turns were washed 3 × 5 min with PBS and mounted with Vectashield with DAPI for nuclear counterstaining (Vector Labs, Burlingame, CA, USA).

Surface preparations of the cochlear turns were observed and photographed under a Zeiss 710 confocal microscope (Zeiss, Oberkochen, Germany). OHC and TUNEL+ cell nuclei counts were carried out, as further detailed.

#### 2.5.2. Immunohistochemistry in Cochlear Sections

Decalcified and washed cochleae were immersed for cryoprotection in 30% sucrose in phosphate buffer (PB) for 48–72 h. Afterwards, the cochleae were embedded in gelatin diluted in 30% sucrose in PB. Gelatin blocks containing the cochleae were quickly frozen at −70 °C using 2-propanol (Sigma-Aldrich) and dry ice and stored at −80 °C. Paramodiolar cochlear Sections 20 mm-thick were obtained using a Leica CM3050 S cryostat (Leica, Wetzlar, Germany). Immunofluorescence detections were performed for the localization of the oxidative stress marker 3-NT and the key oxidative stress enzyme Gpx-1. Antibodies and dilutions are detailed in Table 2.

The cochlear sections, stored at −80 °C, were tempered for 30 min at room temperature and then post-fixed with PFA 4% for 8 min. After washing the slides 3 times for 5 min with PBS, 1 mL of blocking and permeabilization solution (PBS-Tx100 0.25%-BSA 2%) was added, keeping it at room temperature for 1 h. Incubation with the primary antibodies was carried out overnight at 4 °C. The next day, the slides with the sections were washed 3 times for 5 min with PBS and incubation with the secondary antibody was then carried out for 1 h at room temperature in darkness. All antibodies were diluted in PBS-BSA 0.5%. After washing 3 times for 5 min with PBS, the slides were cover slipped with Vectashield (Vector Labs) with DAPI and observed and analyzed in a Zeiss 880 (Zeiss) confocal microscope.

#### 2.5.3. Quantification of OHCs and TUNEL-Positive Nuclei

Quantitative analysis was carried out using ImageJ software (US National Institutes of Health, Bethesda, MD, USA). OHC counts were performed according to previously published protocols [33] adapted to fluorescent labeling of cochlear turns (see above). To quantify OHCs, each labeled cochlear spiral, sectioned in three to four pieces as described above, was photographed under the confocal microscope. Images were orderly arranged in the apical to basal direction. Using ImageJ, 0.1 mm-long segments were delineated through the apical portion of inner hair cells with the software *“segmented line”* tool. In each of these segments, the number of myosin VIIa-immunolabeled OHCs was counted, and the percentage of lost cells per 0.1 mm cochlear length was determined. In addition, the number of TUNEL positive nuclei for every 0.1 mm of organ of Corti’s length was also counted.

#### 2.5.4. Semi-Quantitative Analysis of the Intensity of Immunofluorescence in Cochlear Sections

Photomicrographs were obtained from immunolabeled sections of the cochlea with a confocal fluorescence microscope. Pictures were taken under the same conditions (incubation protocols, confocal settings, software parameters: exposure time, digital zoom, brightness), so that all were comparable to each other in terms of relative fluorescence signal strength. Photomicrographs were analyzed with ImageJ software. The region of interest (OHC region, excluding nuclei, or stria vascularis, excluding blood vessel profiles) was delimited, using ImageJ’s “polygon selections” tool. Once this was achieved, the “mean gray value” was obtained exclusively for the previously delimited area, so that the average intensity of fluorescence in that area, in grayscale, is obtained. Thus, semi-quantitative comparison of relative immunofluorescence intensities between different conditions can be made.

### 2.6. Statistical Analysis

For a comparison of differences of means between groups, one-factor ANOVA was used. In cases where the average of three or more groups was compared and statistical significance was obtained, a Tukey–Kramer post hoc test was performed to determine paired groups between which statistically significant differences existed. Data are expressed as mean±standard deviation (SD.) Statistical significance was defined as * *p* < 0.05, ** *p* < 0.005, *** *p* < 0.001.

## 3. Results

### 3.1. Progression of Hearing Loss with Kanamycin and Cisplatin Injections: ABRs

#### 3.1.1. Kanamycin

**Figure 1:**
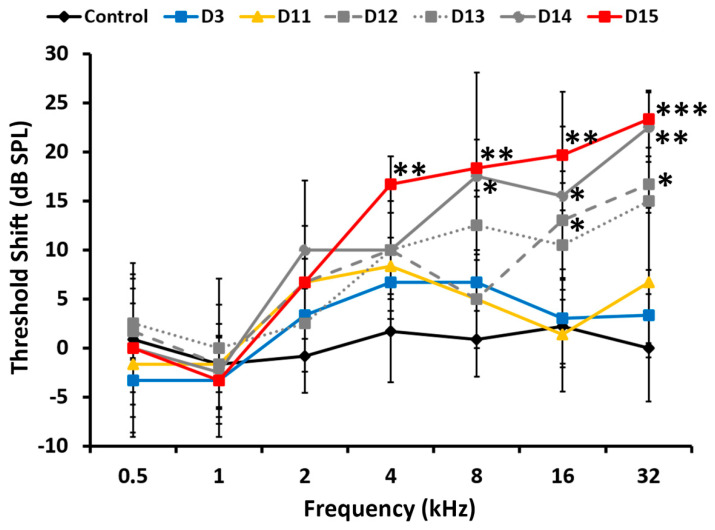
Threshold shift progression in rats treated daily with subcutaneous kanamycin (400 mg/kg/day) for up to 15 days. The progression of threshold shifts is shown at days 3 (D3), 11 (D11), 12 (D12), 13 (D13), 14 (D14) and 15 (D15) after the initiation of treatment. Values are expressed as mean±SD. Significant threshold shifts are first recorded at D12 at 16 kHz and above. At D14, elevated thresholds affect frequencies down to 8 kHz, and by D15 there are elevated thresholds down to 4 KHz. * *p* < 0.05, ** *p* < 0.005, *** *p* < 0.001 vs. control group treated with vehicle.

During treatment with kanamycin, the rats first showed increased auditory thresholds in ABR recordings at day 12 of treatment (D12), starting at the highest frequencies. Significant threshold shifts of 16.67 ± 2.89 dB SPL at 32 kHz (*p* < 0.005) and 13 ± 5 dB SPL at 16 kHz (*p* < 0.05) (Figure 1) were found at D12, compared to the control condition. From D12 to the end of treatment at day 15 (D15), progressively lower frequencies were affected. Thresholds at higher frequency, already increased in previous days of treatment, were also overall higher. However, ototoxicity manifestations of kanamycin observed in ABR recordings showed some variability in initiation times around D12. For instance, at day 13 of treatment (D13), there were threshold shifts of 15 ± 7.07 dB SPL at 32 kHz, 10.5 ± 3.54 dB SPL at 16 kHz and 12.5 ± 3.54 dB SPL at 8 kHz. Although these average threshold shift values were close to those obtained at D12, they did not show statistical significance relative to controls. At day 14 of treatment (D14), there were significant threshold shifts of 22.5 ± 3.54 dB SPL at 32 kHz (*p* < 0.005), 15.5 ± 10.61 dB SPL at 16 kHz (*p* < 0.05) and 17.5 ± 10.61 dB SPL at 8 kHz (*p* < 0.05), relative to the control group (Figure 1). At the end of treatment, on day 15 (D15), there was an extensive alteration of auditory thresholds spanning a broad spectrum of frequencies above 4 kHz. Statistically significant threshold shifts with averages of 23.33 ± 2.89 dB SPL at 32 kHz (*p* < 0.001), 19.67 ± 2.89 dB SPL at 16 kHz (*p* < 0.005), 18.34 ± 2.89 dB SPL at 8 kHz (*p* < 0.005) and 16.67 ± 2.89 dB SPL at 4 kHz (*p* < 0.005), relative to the control group, were consistently recorded.

#### 3.1.2. Cisplatin

**Figure 2:**
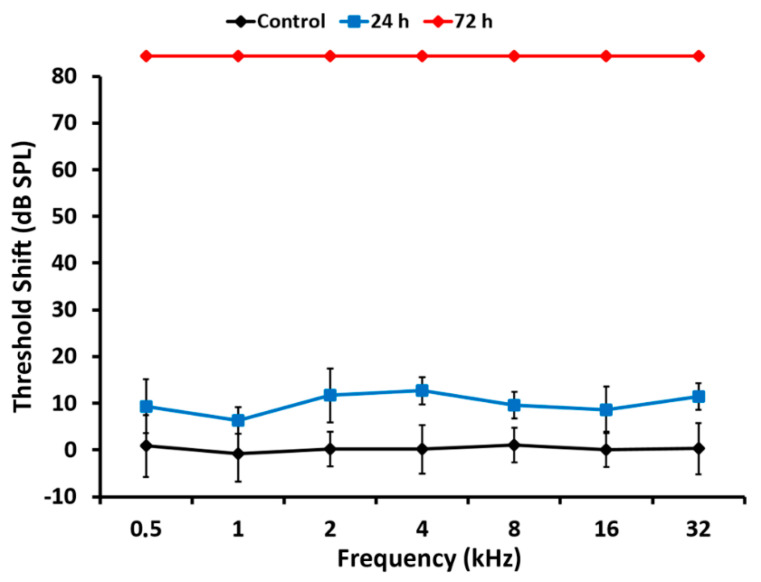
Auditory threshold shifts after 24 h and 72 h of intraperitoneal cisplatin administration (single dose of 16 mg/kg). Relative to control rats, there are slight, statistically non-significant threshold shifts after 24 h of the injection. In contrast, after 72 h, thresholds are not detectable at any tested frequency. Values are expressed as mean ± SD.

ABR recordings performed within 24 h after administration of cisplatin in single injection showed a trend towards elevated thresholds, particularly above 4 KHz. Such threshold shifts, however, did not reach statistical significance at any tested frequency (Figure 2). In contrast, in ABR recordings performed 72 h after cisplatin administration, there was a total absence of recordable auditory evoked potentials at all frequencies and intensities, with thresholds becoming undetectable (Figure 2). Therefore, under our experimental conditions, cisplatin-mediated hearing loss progresses from slight, statistically not significant threshold elevations at 24 h to thresholds becoming undetectable at 72 h after administration.

### 3.2. Temporal and Spatial Sequence of OHC Loss after Ototoxic Kanamycin or Cisplatin Treatment

#### 3.2.1. Kanamycin

**Figure 3:**
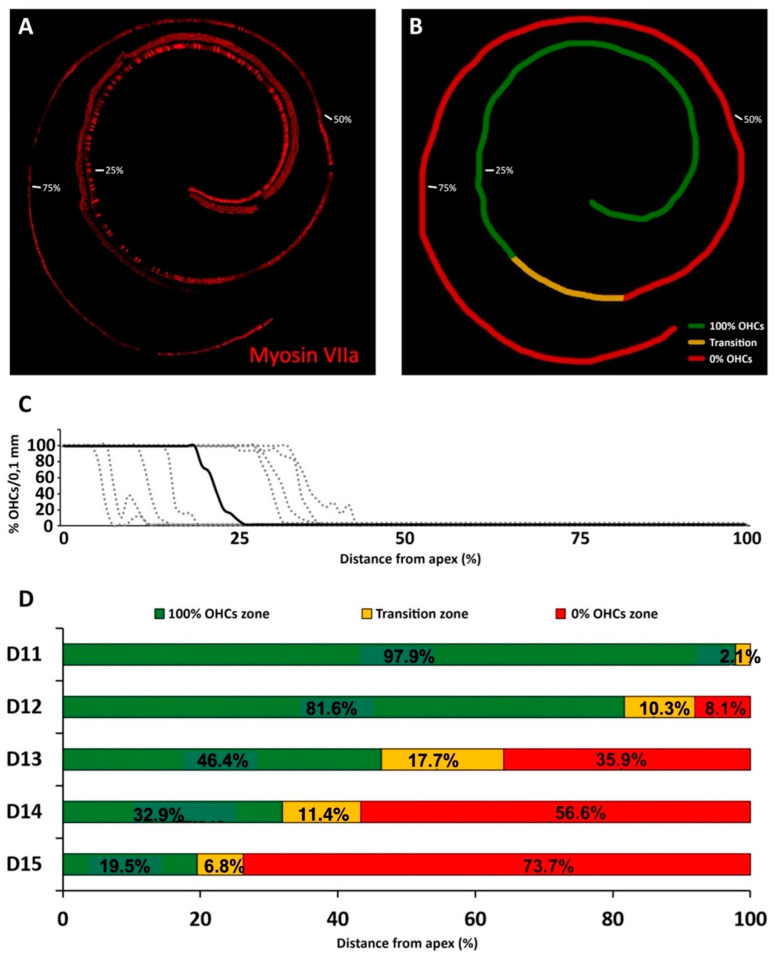
(**A**): Reconstruction of a representative cochlear spiral, immunostained by immunofluorescence with myosin VIIa, from the cochlea of a rat treated with kanamycin (400 mg/kg/day) at D15 of treatment. (**B**): Color-coded schematic representation of the cochlear spiral in (**A**). The segment with complete preservation of OHCs, the transition zone and the zone with complete loss of OHCs are represented, respectively, in green, yellow and red. Percent values in (**A**,**B**) indicate relative cochlear distance starting at the apex. (**C**): OHC counts from surface preparations of the organ of Corti at the end of treatment with kanamycin, at D15. The percentage OHC loss/0.1 mm length is charted along the organ of Corti. Each dotted line is an individual animal. The solid line is the average. (**D**): Temporal and spatial sequence of OHC loss with kanamycin. Changes with time after treatment are represented as the mean percentage of the length of the organ of Corti occupied by the zone with full preservation of OHCs (green), the transition zone (yellow) and the zone with complete loss of OHCs (red) in rats treated with kanamycin at D11, D12, D13, D14 and D15. Note that the length of the transition zone, a potential surrogate of OHC death rate, is maximum at D13 and declines sharply by D15.

Cell counts on surface preparations of cochlear turns from rats treated daily with kanamycin at 400 mg/kg bw for up to 15 days, showed complete preservation of OHCs in variable lengths of the apical portion of the organ of Corti, which depended on the day of treatment analyzed (Figure 3). In contrast, complete loss of OHCs was recorded in variable lengths of more basal turns (Figure 3A–D). Between these two segments, there was an intervening third (Figure 3 D), designated as a “*transition zone”* to stress the notion that active processes of apoptotic cell death are taking place, as reported below, and are progressively “transitioning” towards a complete disappearance of OHCs. There was no significant loss of inner hair cells (IHCs) in any region of the organ of Corti (Figure 3A).

A detailed quantitative analysis of the progression of OHC loss caused by kanamycin showed that it is first detectable at D11, with cochleae at D3 undistinguishable from controls. At D11, the stretch containing mostly preserved OHCs represented a total apical-to-basal length of 97.9 ± 3.7% of the organ of Corti. A short transition zone occupied 2.1 ± 3.7%, at the most basal end of the total length of the organ of Corti (Figure 3D).

Loss of OHCs progressed continuously from D12 onwards (Figure 3D). At this treatment day, the most basal segment with a total loss of OHCs occupied 8.1 ± 9.8% of the total length, and the segment with preservation of OHCs occupied 86.1 ± 10.8% of the total length, starting at the apical end of the of the organ of Corti (Figure 3D). The intervening transition zone occupied an average of 10.3 ± 1% of the total length (Figure 3D).

The cochleae of rats at D13 showed an apical stretch of the organ of Corti with total preservation of OHCs representing an average of 46.4 ± 5.9% of the total length of the receptor lamina. The basal segment showing a total loss of OHCs was 35.9 ± 13.6% of the total length. The transition zone in-between represented 17.7 ± 7.8% of the total length (Figure 3D).

At D14, the segment with seemingly intact OHCs occupied an average length of 32 ± 12.6%, starting at the apical end of organ of Corti, whereas the total absence of OHCs was recorded in 56.6 ± 16.2% of the length, starting at the basal end. The transition zone was 11.4 ± 3.7% of the total length (Figure 3D).

At the end of treatment on D15, there was complete preservation of OHCs in the most apical 19.5 ± 11% of the total length of the organ of Corti. The total loss of OHCs was recorded in an average length of 73.7 ± 12.5% towards the basal end (Figure 3C,D). The transition zone represented at this end point of treatment was 6.8 ± 2.8% of the total length of the organ of Corti (Figure 3D).

#### 3.2.2. Cisplatin

**Figure 4:**
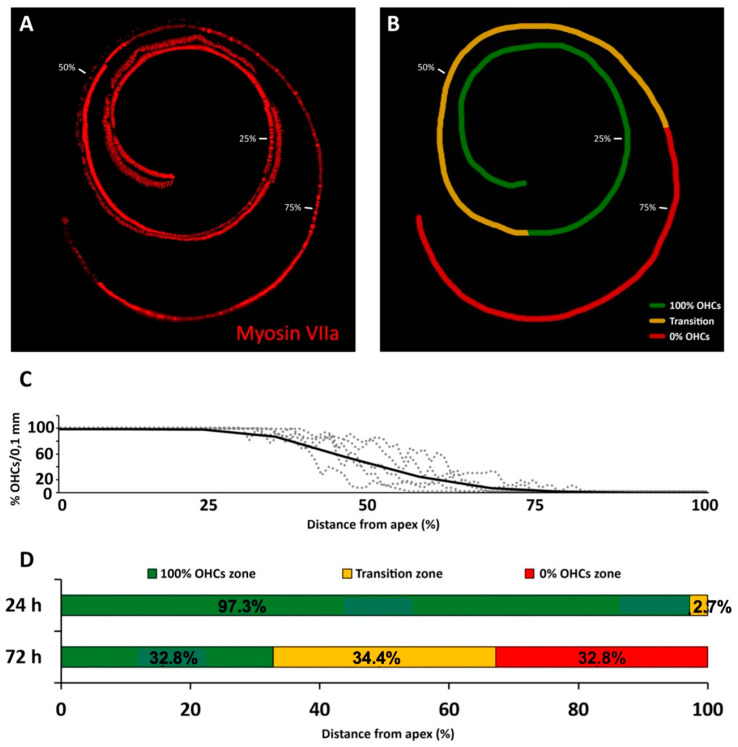
(**A**): Reconstruction of a representative cochlear spiral, immunostained by immunofluorescence with myosin VIIa, from the cochlea of a rat treated with a single injection of cisplatin (16 mg/kg bw) at 72 h after administration of the drug. (**B**): Color-coded schematic representation of the cochlear spiral in (**A**). The segment with complete preservation of OHCs, the transition zone and the zone with complete loss of OHCs are shown, respectively, in green, yellow and red. Percent values in (**A**,**B**) indicate relative cochlear distance starting at the apex. (**C**): OHC counts from surface preparations at the end of treatment with cisplatin, at 72 h. The percentage of OHC loss/0.1 mm length is charted along the complete extension of the organ of Corti. Each dotted line is an individual animal. The solid line is the average. (**D**): Temporal and spatial sequence of OHC loss with cisplatin. Changes with time after treatment represented as the mean percentage of the length of the organ of Corti occupied by the zone with full preservation of OHCs (green), the transition zone (yellow) and the zone with complete loss of OHCs (red) in rats treated with cisplatin, 24 and 72 h after initiation of treatment.

OHC counts on surface preparations (Figure 3A,B) of cochlear turns obtained from rats 24 h after administration of cisplatin showed an apical segment with no detectable loss of OHCs, comprising 97.3 ± 5.4% of the total length of the organ of Corti. A transition zone was identified at the cochlear base, comprising 2.7 ± 5.4% of the total length (Figure 4D). There was no detectable loss of IHCs.

Cochlear turns from rats 72 h after administration of cisplatin (Figure 4A–D) showed an OHC loss pattern (Figure 4C,D), also characterized by three clearly differentiated regions (Figure 4A,D) or segments. The apical segment, with complete preservation of OHCs, occupied on average 32.8 ± 11.3% of the total length, the area with complete loss of OHCs in basal regions corresponded with an average length of 32.8 ± 11.3% and the transition zone located between the previous two zones represented an average of 34.4 ± 11.8% of the total length (Figure 4D).

### 3.3. Spatial Pattern of Apoptosis after Kanamycin or Cisplatin Ototoxicity: TUNEL

#### 3.3.1. Kanamycin

**Figure 5:**
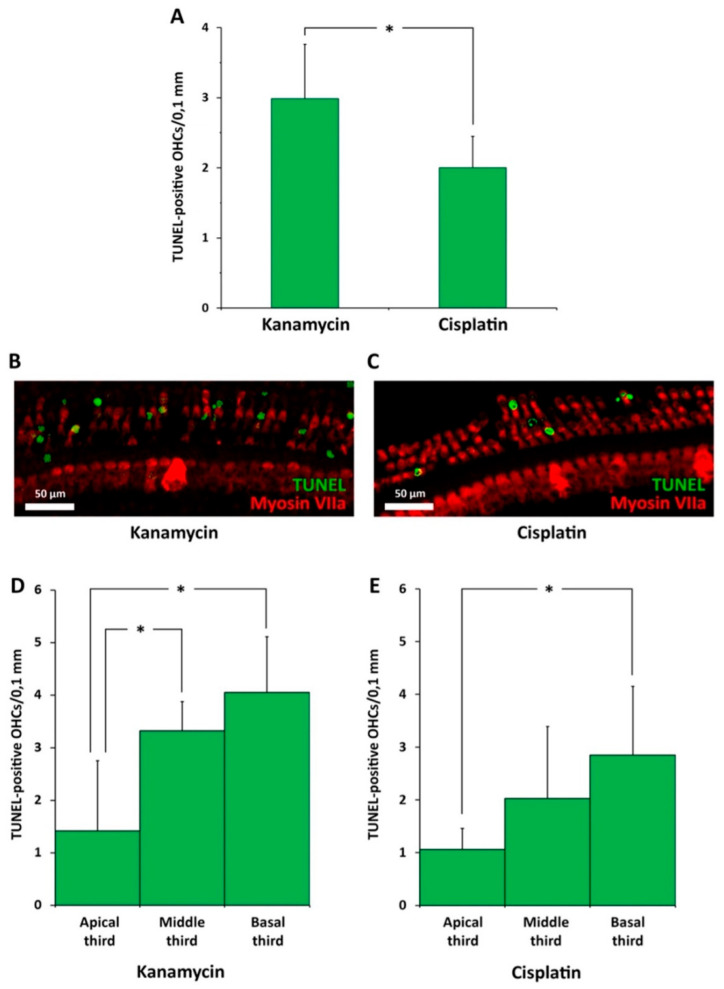
Comparison of apoptosis after kanamycin and cisplatin ototoxicity with the TUNEL method in the transition zone. (**A**): Average number of apoptotic nuclei/0.1 mm length in the transition zone in rats treated with kanamycin (D15) and cisplatin (72 h). (**B**): Apoptotic, TUNEL^+^ nuclei present in the transition zone after treatment with kanamycin (D15). (**C**): TUNEL^+^ nuclei in the transition zone after treatment with cisplatin (72 h). (**D**): Average apoptotic nuclei/0.1 mm length present in each of three thirds of the transition zone after treatment with kanamycin at D15. (**E**): Average number of apoptotic, TUNEL^+^ nuclei/0.1 mm length present in each third of the transition zone, 72 h after treatment with cisplatin. The denomination “apical,” “middle” and “basal” third is in relation to the length of the transition zone and not to cochlear turns. Asterisks indicate statistical significance values, as specified in Section 2.6, Materials and Methods.

At the end of the kanamycin treatment at D15, the TUNEL method showed the presence of apoptotic nuclei (Figure 5A,B) concentrated in the transition zone. The average number of TUNEL^+^ nuclei was 2.99 ± 0.78/0.1 mm (Figure 5A). Within the transition zone, the distribution of TUNEL^+^ nuclei supported spatially patterned apoptotic cell death. Counts of TUNEL^+^ nuclei in three arbitrarily defined segments of equal length in the transition zone demonstrated that the more apical third of the transition zone had a significantly lower number of TUNEL^+^ nuclei (1.41 ± 1.34/0.1 mm) than the intermediate third (3.32 ± 0.56/0.1 mm, *p* < 0.05) and the basal third (4.04 ± 1.07/0.1 mm, *p* < 0.05); (Figure 5D).

#### 3.3.2. Cisplatin

Such as with kanamycin, TUNEL showed the presence of apoptotic nuclei belonging to OHCs located in the transition zone (Figure 5A,C). The average number of TUNEL^+^ nuclei was 2 ± 0.45/0.1 mm length of the organ of Corti. TUNEL^+^ nuclei were undetectable in any of the other two regions differentiated in the organ of Corti, i.e., the segment with preserved OHCs, and the segment with complete loss of OHCs. The number of TUNEL^+^ nuclei found in the transition zone was, on average, significantly lower with cisplatin treatment than with kanamycin (Figure 5A,B). Like in the case of kanamycin, the most basal third of the transition zone showed a significantly higher number of TUNEL^+^ nuclei (2.85 ± 1.29/0.1 mm) than the more apical third (1.06 ± 0.39/0.1 mm, *p* < 0.05); (Figure 5D,E).

### 3.4. 3-NT Immunolabeling: Oxidative Stress in the Cochlea after Kanamycin and Cisplatin Ototoxicity

#### 3.4.1. Kanamycin

**Figure 6:**
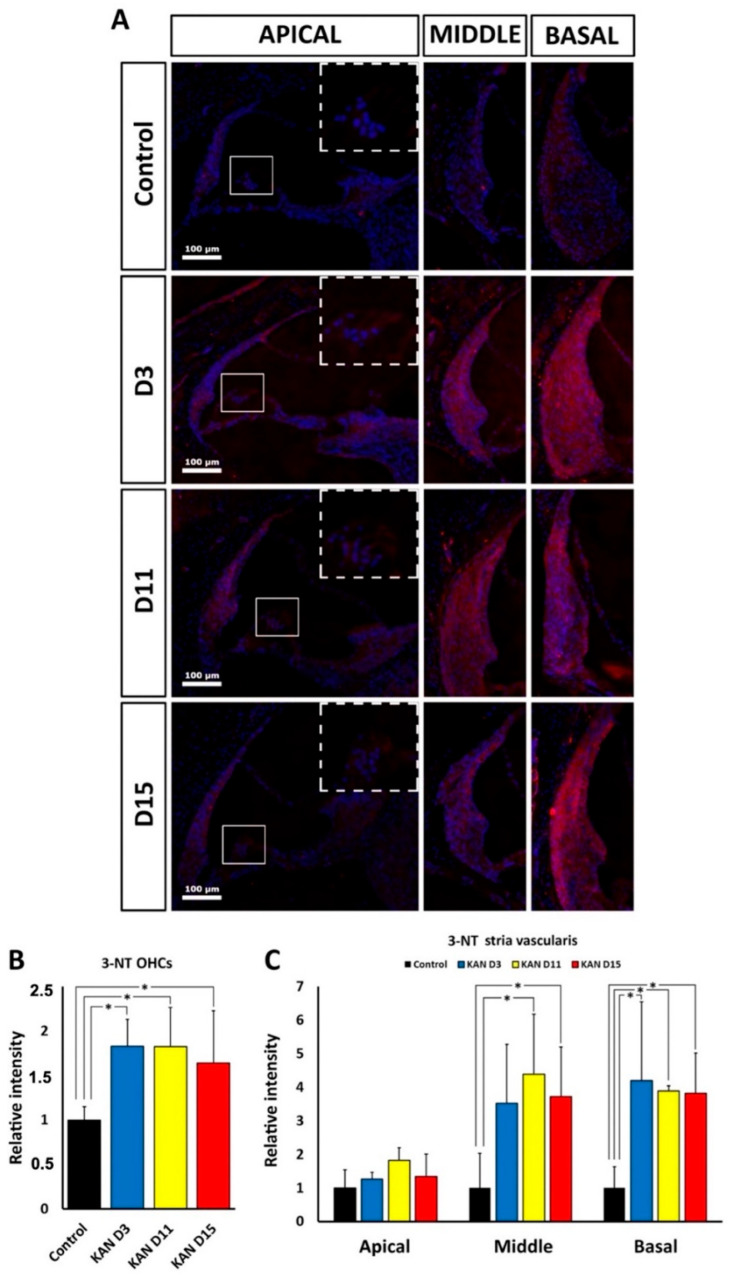
(**A**): 3-NT immunolabeling (magenta) at D3, D11 and D15 after kanamycin (KAN) ototoxic treatment and corresponding control. Nuclei are counterstained with DAPI (blue). In the first vertical panel, at the right of the figure, the square highlights the OHC region in the apical turn, which is enlarged in the square delimited with a broken line at the different survival times after treatment with kanamycin and in control animals. The lateral wall in the cochlear apex can also be observed. The next two panels show 3-NT immunolabeling in the lateral wall, including the stria vascularis of the middle and basal turns during treatment with kanamycin, at D3, D11 and D15 and in control animals. (**B**): Relative intensity of 3-NT immunolabeling in the OHC region of the apical turn during treatment with kanamycin, at D3, D11 and D15. (**C**): Relative intensity of 3-NT immunolabeling in the stria vascularis of the apical, middle and basal turns during treatment with kanamycin, at D3, D11 and D15. Asterisks indicate statistical significance values, as specified in Section 2.6, Materials and Methods.

To test “in situ” oxidative stress levels in cochlear structures after kanamycin ototoxicity, immunofluorescence was performed using an anti-3-NT antibody to detect nitrosylated products of oxidation. A semi-quantification of fluorescence signal intensity was carried out as described in the Materials and Methods section. There was a statistically significant increase in the intensity of NT-3 immunolabeling relative to the control group in preserved OHC regions of the apical turn at D3 (*p* < 0.05), D11 (*p* < 0.05) and D15 (*p* < 0.05; Figure 6A,B). In the stria vascularis, 3-NT immunolabeling intensity also increased significantly relative to controls, at D3 (*p* < 0.05), D11 (*p* < 0.05) and D15 (*p* < 0.05) in the basal turn (Figure 6A,C), and at D11 (*p* < 0.05) and D15 (*p* < 0.05) in the middle turn. However, there were no significant NT-3 immunolabeling intensity variations in apical parts of the stria vascularis at any time point of the treatment (Figure 6C).

#### 3.4.2. Cisplatin

**Figure 7:**
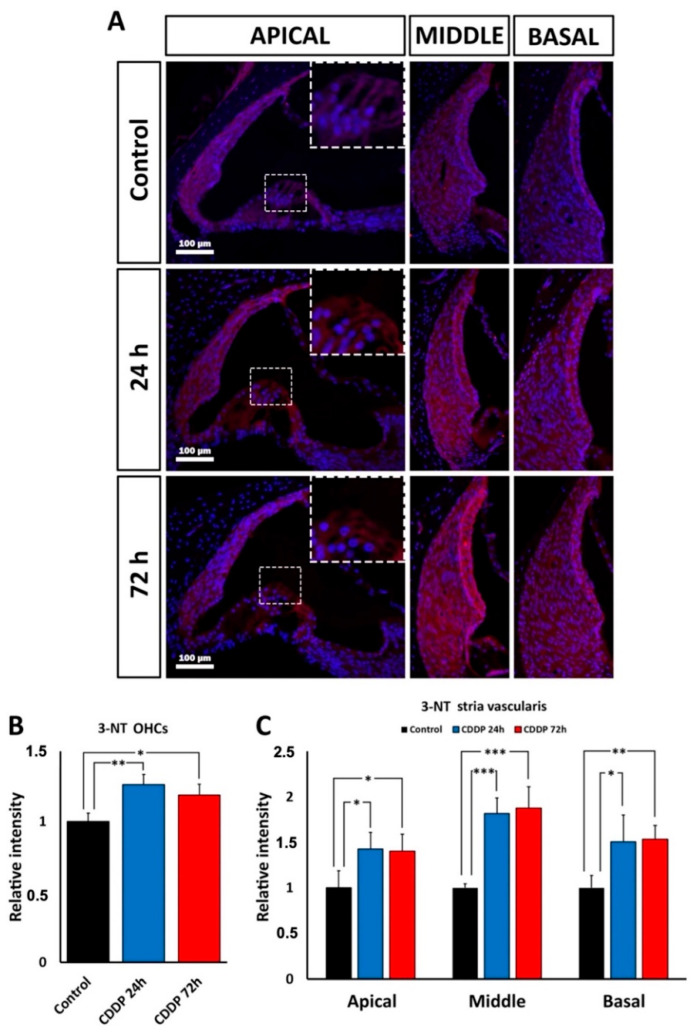
(**A**): Fluorescent immunolocalization of 3-NT (magenta) in the cochlea after ototoxic treatment with cisplatin (CDDP) and corresponding untreated control. Nuclei are counterstained in blue with DAPI. The vertical panel at the right of the illustration shows immunolabeling for 3-NT in the OHC region of the apical turn (small square, enlarged in the upper left corner of the photomicrography). The stria vascularis of the apical region can also be observed. The next two vertical panels show the lateral wall, including the stria vascularis of the middle and basal turns after 24 h and 72 h of cisplatin administration. Details are given in the text. (**B**): Relative immunolabeling intensity of 3-NT in the OHC region of the apical turn, 24 h and 72 h after administration of cisplatin. (**C**): Relative intensity of 3-NT immunolabeling in the stria vascularis of the apical, middle and basal turns, 24 h and 72 h after administration of cisplatin. Asterisks indicate statistical significance values, as specified in Section 2.6, Materials and Methods.

With cisplatin, there was also a significant increase in relative intensity levels of 3-NT immunolabeling in preserved OHC regions, compared to the control group, at 24 h (*p* < 0.005) and at 72 h (*p* < 0.05) of cisplatin administration (Figure 7A,B). 3-NT immunoreactivity levels also were significantly increased in the stria vascularis at 24 h of drug administration in the apical (*p* < 0.05), middle (*p* < 0.001) and basal turns (*p* < 0.05), and at 72 h also in the apical (*p* < 0.05), middle (*p* < 0.001) and basal turns (*p* < 0.005); (Figure 6A–C).

### 3.5. Antioxidant Enzyme Genes GPx1, Cat and SOD1 after Kanamycin and Cisplatin Ototoxicity

#### 3.5.1. Changes in the Expression of GPx1, Cat and Sod1 Genes after Kanamycin Treatment

**Figure 8:**
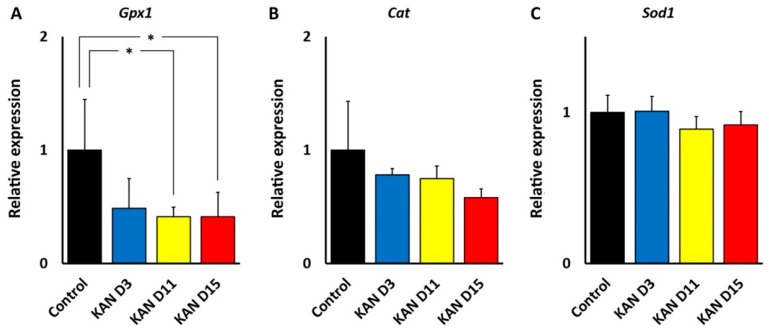
Changes in the expression of antioxidant enzyme genes in the cochlea after treatment with kanamycin (KAN). Relative expression level of the Gpx1 (**A**), Cat (**B**) and Sod1 (**C**) genes under conditions of kanamycin ototoxicity at D3, D11 and D15. See text for details. Asterisks indicate statistical significance values, as specified in Section 2.6, Materials and Methods.

As far as genes coding for three main antioxidant enzymes (Gpx1, Cat and Sod1) are concerned, RT qPCR showed that treatment with kanamycin induced a significant decrease in the expression of Gpx1 at D11 (fold change, 0.38; *p* < 0.05) and D15 (fold change, 0.38; *p* < 0.05), relative to the control group. Decreased expression (fold change, 0.45) was also detected at D3, although it did not reach statistical significance (Figure 8A). The Cat gene appeared to decrease its expression from the beginning relative to the control group, although such a decrease lacked statistical significance (fold change 0.80, 0.76 and 0.57 respectively at D3, D11 and D15) (Figure 8B). Finally, no changes in gene expression for Sod1 were observed at any time point after treatment with kanamycin (fold change 0.98, 0.86 and 0.91, respectively, at D3, D11 and D15) (Figure 8C).

#### 3.5.2. Changes in the Expression of GPx1, Cat and Sod1 Genes after Cisplatin Treatment

**Figure 9:**
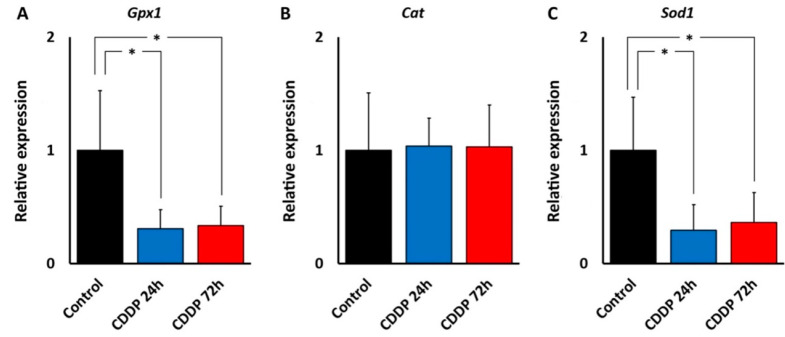
Changes in the expression of antioxidant enzyme genes in the cochlea with cisplatin treatment (CDDP). A: Relative expression levels of the Gpx1 (**A**), Cat (**B**) and Sod1 (**C**) genes under conditions of cisplatin ototoxicity at 24 h and 72 h after injection. See text for details. Asterisks indicate statistical significance values, as specified in Section 2.6, Materials and Methods.

RT-qPCR revealed a strong, statistically significant decrease in the relative expression level of the Gpx1 gene in the cochlea after 24 h (fold change, 0.28; *p* < 0.05) and 72 h (fold change, 0.33; *p* < 0.05) of cisplatin administration, relative to the control group (Figure 9A). In contrast, the expression of the Cat gene did not change at any time point tested (fold change, 1.04 and 1.03, respectively, at 24 and 72 h) (Figure 9B). On the other hand, the expression of the Sod1 gene experienced a marked, significant decrease at 24 h (fold change, 0.27; *p* < 0.05) and 72 h (fold change, 0.31; *p* < 0.05) of cisplatin treatment, relative to the control group (Figure 9C). Asterisks indicate statistical significance values, as specified in Section 2.6, Materials and Methods.

### 3.6. GPx1 Immunoreactivity in the Cochlea after Kanamycin and Cisplatin Treatment

Based on the changes in gene expression described above, we tested differences in Gpx1 immunolabeling for kanamycin and cisplatin. Gpx1 was selected because its expression is downregulated both after kanamycin and cisplatin treatment.

#### 3.6.1. Gpx1 Immunoreactivity after Kanamycin Ototoxicity

**Figure 10:**
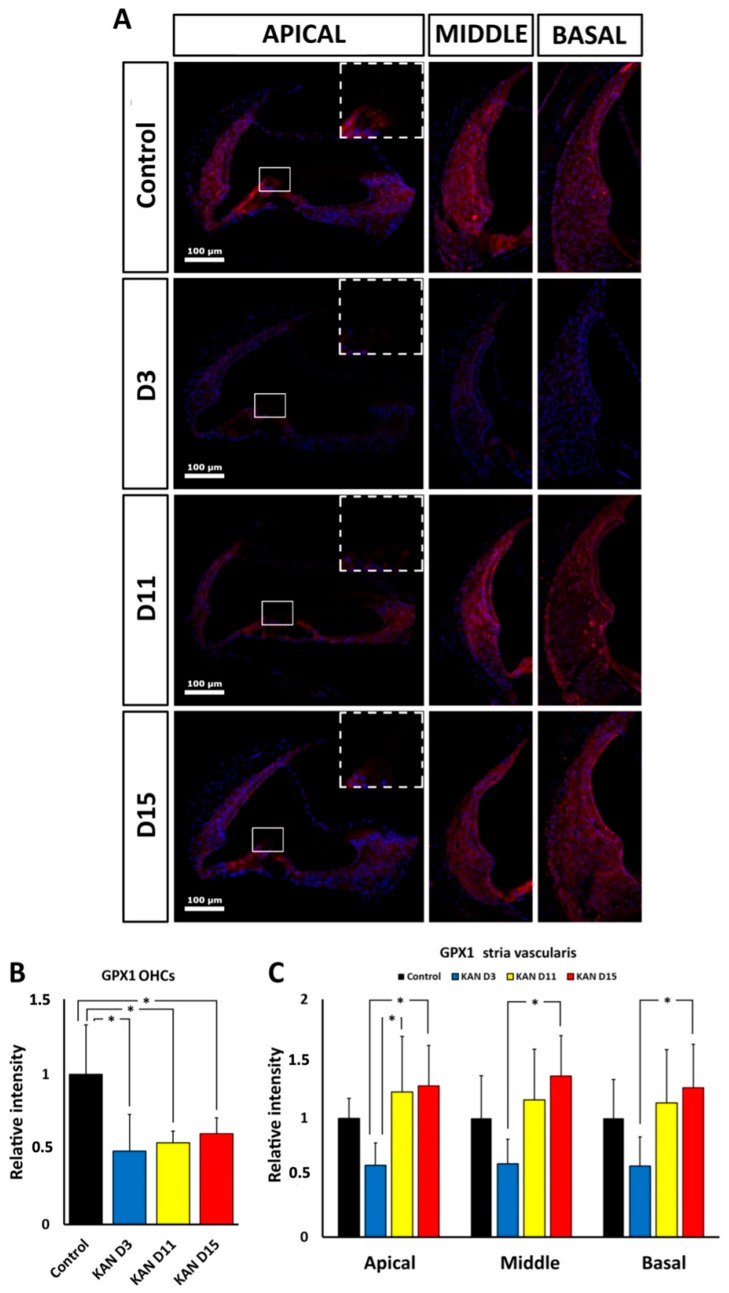
(**A**): Fluorescent immunolocalization of GPx1 (magenta) in the cochlea after kanamycin (KAN) ototoxicity and corresponding untreated control. Nuclei are counterstained with DAPI (blue). The first vertical panel at the right of the figure shows the OHC region of the apical turn (square, enlarged in the upper left corner of the photomicrograph). The lateral wall region with the stria vascularis can also be observed. The next two vertical panels show the lateral wall, including the stria vascularis of the middle and basal turns at D3, D11 and D15 after kanamycin treatment. (**B**): Relative intensity levels of GPx1 in the OHC region of the apical turn during treatment with kanamycin, at D3, D11 and D15. (**C**): Relative intensity levels of GPx1 immunolabeling in the stria vascularis of the apical, middle and basal turns during ototoxic treatment with kanamycin, at D3, D11 and D15. At D11 and D15, relative intensity values are statistically not significantly different to those measured in controls. Asterisks indicate statistical significance values, as specified in Section 2.6, Materials and Methods.

Semiquantitative immunocytochemical localization showed a significant decrease in GPx1 immunolabeling in the OHC region, relative to controls, at D3 (*p* < 0.05), D11 (*p* < 0.05) and D15 (*p* < 0.05; Figure 10A,B). Diminished immunolabeling levels of GPx1 at D3 were observed in the apical, middle and basal regions of the stria vascularis. However, the measurement of relative immunolabeling intensity levels showed no statistically significant differences relative to controls (Figure 10A,C). Likewise, at D11 and D15, GPx1 immunoreactivity intensity levels in the stria vascularis were not significantly increased, relative to controls, although, relative to D3, the labeling intensity was higher at D11 in the apical turn (*p* < 0.05) and at D15 in the apical (*p* < 0.05), middle (*p* < 0.05) and basal turns (*p* < 0.05); (Figure 10A,C).

#### 3.6.2. Gpx1 Immunoreactivity after Cisplatin Ototoxicity

**Figure 11:**
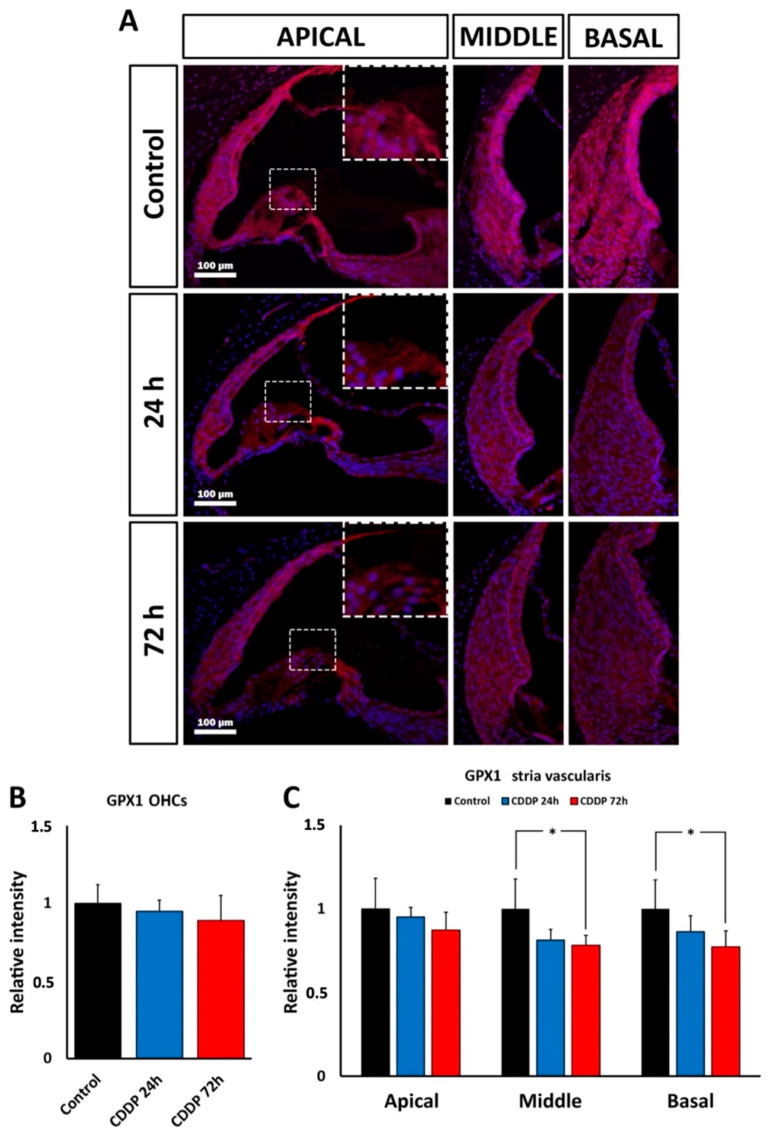
(**A**): Fluorescence immunolabeling for GPx1 (magenta) in the cochlea after 24 and 72 h of ototoxic cisplatin (CDDP) treatment and corresponding untreated control. Nuclei are counterstained with DAPI (blue). The vertical panel at the right of the figure shows immunolabeling for GPx1 in the OHC region of the apical turn (square, enlarged in the inset limited by broken line). The lateral wall with the stria vascularis can also be observed. The adjacent two panels show the lateral wall, including the stria vascularis of the middle and basal turns after 24 h and 72 h of cisplatin administration. (**B**): Relative intensity of GPx1 immunolabeling in the OHC region of the apical turn, 24 h and 72 h after administration of cisplatin. (**C**): Relative intensity of GPx1 immunolabeling in the stria vascularis of the apical, middle and basal turns, 24 h and 72 h after cisplatin administration. Asterisks indicate statistical significance values, as specified in Section 2.6, Materials and Methods.

Immunocytochemical labeling showed no significant changes in relative intensity levels of GPx1 in the OHC region at any time point relative to the control group (Figure 11A,B). In the stria vascularis, however, there was a decrease in GPx1 immunolabeling levels, especially 72 h after treatment, which was statistically significant in the middle (*p* < 0.05) and basal (*p* < 0.05) turns; (Figure 11A,C).

## 4. Discussion

We report results of a side-by-side comparison of two commonly used experimental protocols reproducing kanamycin and cisplatin ototoxicity in the rat [33,34]. The metrics included auditory threshold shifts in ABRs, in correlation with rates of OHC loss and spatial patterns of apoptosis, along with the involvement of oxidative stress and enzymatic oxidative “defenses” in cochlear damage mediated by these two drugs. These may be relevant to interpret future therapeutical outcomes, in particular with antioxidation therapies.

For kanamycin, the first statistically significant threshold increase, around 15 dB, was observed at days 12 or 13 of treatment, at the highest frequencies tested of 16 kHz and 32 kHz. At day 14, threshold shifts increased further, up to 23 dBs, at these same frequencies. The 8 kHz frequency was also affected, with threshold shifts over 15 dB. At day 15, threshold shifts were even more evident in the same frequency range, and extended further down to 4 kHz, where threshold shifts of over 15 dB were recorded. Loss of hearing sensitivity centered initially at high frequencies and progressively extending to lower frequencies is a landmark of aminoglycoside ototoxicity [1,33,35]. Our results demonstrate that such hearing loss in the Wistar rat concentrates in the last four days of treatment, progressing quickly from higher to lower frequencies down to 4 kHz.

High frequency threshold elevations at day 12 after treatment corresponded with missing OHCs in the most basal 8.1% of the total cochlear length. An adjoining stretch in which ongoing apoptosis was detectable with the TUNEL method, the “transition zone,” spanned an average of 10.3% of cochlear length. Therefore, by the time the first significant auditory threshold elevations were recorded during kanamycin treatment, 18.4% length of the cochlear neuroepithelium was damaged, as judged by the total or partial loss of OHCs. Coincident with threshold shifts, from day 12 to day 14 after kanamycin treatment there was a fast progression of OHC loss. The average percent cochlear length showing OHC absence at day 14 (56.6%) was almost 6.7 times larger than at day 12 (8.1%). Threshold elevations were correspondingly larger and spanned a wider tested frequency range, down to 8 KHz. By day 15, the last kanamycin post-treatment timepoint assessed, the absence of OHCs affected 73.9% of the cochlear length starting at the basal end, which corresponded with increased thresholds at frequencies down to 4 kHz. Although the end of kanamycin treatment at day 15 is somehow arbitrary, it has been demonstrated that at doses of 300 to 500 mg/kg in the rat, OHCs in the cochlear apex are largely preserved even after three weeks of the end of a 15-day treatment [33].

The fast progression of the cochlear length affected by complete loss of OHCs was paralleled by a progressive decrease in the length of the adjoining transition zone, from 17.3% at day 13 to 6.3% at day 15. This suggests that apoptotic cochlear cell death mediated by kanamycin evolves on a spatially ordered fashion starting at the basal end, as shown by the identification of a transition zone, with undergoing nuclear DNA fragmentation, as detected by the TUNEL method. As ototoxicity progresses, the final stages of apoptosis downstream of DNA fragmentation, therefore not detectable with TUNEL, may predominate. Consequently, more length of the cochlear spiral will be contained within the region devoid of OHCs. In this regard, the reported quick shortening of the transition zone to a length of just 6.3% of the cochlear spiral at day 15 post-kanamycin treatment, may anticipate the spatial “fading away” of kanamycin-mediated OHC apoptosis towards the cochlear apex. At this endpoint of kanamycin treatment, the most apical 19.5% end of the cochlea was preserved. Because the cochlear span showing apoptosis with TUNEL labeling shortens dramatically between days 13 and 15 after treatment, regardless of the participation of other cell death pathways such as necroptosis [19], it is conceivable that such a 19.5% length of cochlear apex at day 15 post-treatment was relatively better preserved [33], representing a relevant functional region of “residual hearing” [36], tuned approximately at 2 to 0.5 kHz in the rat.

As expected, the loss of hearing sensitivity with a single subcutaneous injection of cisplatin at 16 mg/kg differed in pattern from that of kanamycin. Within 24 h after the injection, there was no significant threshold shift in ABRs at any tested frequency. Seventy-two hours after the injection, however, evoked activity that was not recordable, precluding accurate threshold determination at any tested frequency. High threshold elevations across the entire frequency range, in occasions at the limit of precise determination, have been reported previously with comparable treatment protocols [37]. Such extreme loss of hearing sensitivity facilitates further insights in relation with the corresponding OHC loss. After 24 h of cisplatin administration, when ABR recordings are still within normal range, OHCs were present on 97.3% of the cochlear length, with the most basal 2.7% showing apoptosis. These do not differ much to those found with kanamycin after 11 days of treatment, supporting similarities for both drugs in the sensitivity of the receptor lamina at the outset of ototoxicity. However, 72 h after the administration of cisplatin, when auditory evoked potentials were no longer recordable, roughly the most apical third of the receptor lamina length was preserved as far as OHCs are concerned, whereas the most basal third concentrates OHC loss. In the intervening third, the transition zone, TUNEL^+^ nuclei coexisted with recognizable OHCs. Such as with kanamycin, counts of TUNEL^+^ nuclei suggest that cell death progressed in a spatially ordered fashion from base to apex. Moreover, comparatively lower numbers of TUNEL^+^ nuclei with cisplatin suggest different rates of ototoxic cell death.

In sum, after 72 h of cisplatin administration, there was OHC preservation in 35% of the length receptor lamina at its apical end with no recordable ABRs. This is in sharp contrast with kanamycin; at the 15 day-endpoint of treatment, as already discussed, 19.5% of the apical end contained preserved OHCs, with normal auditory thresholds in the 0.5–2 kHz range. The percent length of the preserved auditory neuroepithelium in the cochlear apex at the end of cisplatin treatment was 1.6 times larger than with kanamycin. Thus, the complete loss of hearing sensitivity with high doses of cisplatin does not necessarily involve a massive loss of receptor cells. Even with approximately 1/3 of the cochlear length containing identifiable OHCs at the endpoint of cisplatin treatment, there was no recordable activity in ABRs. This supports that a considerable fraction of OHCs may survive immediately after cisplatin ototoxicity but are not able to generate receptor potentials. As discussed further, this may be because, as compared with kanamycin, the structure and function of the stria vascularis, involved in ion concentration homeostasis necessary for the electrical activity of sensory receptor cells, is more compromised with cisplatin [10,21,38,39]. With this cisplatin ototoxicity protocol, it is not possible to know the final fate of the surviving OHCs past day three of treatment, despite which it seems safe to conclude that their function is compromised from early stages of ototoxicity. The fact that they are still structurally preserved may have implications for establishing a recovery window.

In this regard, the immunohistological distribution of the oxidative stress marker 3-NT provides additional information about possible differences in ototoxic mechanisms and progression between kanamycin and cisplatin. 3-NT accumulates because of extensive nitrosylation of tyrosine by excess nitrogen free radicals, mainly peroxynitrites. This renders structurally and functionally altered proteins in the chain of events leading to oxidative damage. 3-NT immunolabeling was detectable in the cochlea, particularly in the organ of Corti and lateral wall structures, both after kanamycin and cisplatin treatment. This supports oxidative stress mediating ototoxicity by both drugs, as previously reported [4,6,8,10,14,15]. However, we found different cochlear distribution of 3-NT immunolabeling, pointing to differences in mechanisms involving oxidative stress. It is important to point out that no conclusions can be drawn from comparing relative differences in 3-NT cochlear immunolabeling intensities *between* kanamycin and cisplatin, mainly because antibody incubation times and controls were optimized for each ototoxic treatment, thus limiting the interpretation of such comparisons between both drugs. However, the regional or spatial distribution of cochlear 3-NT immunolabeling intensity can be tested individually for each ototoxic drug and compared. We found that relative 3-NT immunolabeling intensity distribution differs regionally between kanamycin and cisplatin, at least in the stria vascularis. For kanamycin, relative levels of 3-NT immunofluorescence intensity were increased in regions where OHCs could still be identified based on topographical localization of nuclei. In these regions, 3-NT immunolabeling at 3, 11 and 15 days after treatment was significantly more intense than in controls. In the stria vascularis, kanamycin treatment also resulted in increased 3-NT immunolabeling levels, but with noticeable regional differences. Basal regions showed increased 3-NT immunolabeling at all post-treatment times analyzed. In middle regions, significantly elevated 3-NT immunolabeling intensity was detected at 11 and 15 days after treatment. In contrast, in apical regions there was no detectable increase in 3-NT immunolabeling relative to controls at any time post-treatment. Therefore, the apical portions of the stria vascularis seem to be comparatively less sensitive to oxidative stress damage induced by kanamycin. In the case of cisplatin, such as with kanamycin, regions of interest with identifiable OHCs in the cochlear apex also had increased 3-NT immunolabeling throughout treatment time points. In contrast to kanamycin, however, in the stria vascularis increased levels of 3-NT immunolabeling were observed in all turns, both at 24 and 72 h of cisplatin administration. This supports that, different to kanamycin, the whole stria vascularis is overall more sensitive to oxidative stress damage mediated by cisplatin. Long term accumulation of cisplatin has been reported in the stria vascularis [39]. Therefore, the extreme loss of hearing sensitivity observed with cisplatin may be related to massive oxidative damage throughout the stria vascularis. This results in the inability to keep ion concentrations in the endolymph, particularly K^+^, within the range needed to preserve the endocochlear potential, with subsequent blockade of evoked activity, even with a considerable fraction of preserved OHCs at the end of treatment, in comparison with kanamycin.

Thus, regional cochlear differences in susceptibility to oxidative stress may be responsible for differences in the ototoxicity profiles of kanamycin and cisplatin. The central position of oxidative stress in ototoxicity mechanisms is also observed in the expression and localization of enzymes involved in “antioxidation defense.” We found that, in the cochlea, both kanamycin and cisplatin systemic treatment changed the expression levels of genes coding for antioxidation enzymes, with relevant differences between both ototoxic drugs, as observed with qRT-PCR. With kanamycin, mRNA expression levels for Gpx1 dropped significantly after 11 days of treatment and remained diminished at 15 days, whereas mRNA levels for Cat and Sod1, the two other key enzymes for antioxidation, remained unchanged. Down-regulation of Gpx1 in the cochlea after kanamycin treatment has been reported [25]. Different to kanamycin, however, the cisplatin treatment resulted in diminished levels of Gpx1 *and* Sod1 gene expression. Down-regulation of the Sod1 gene with cisplatin but not with kanamycin suggests differences in antioxidation response mechanisms between both ototoxic drugs. On the other hand, Cat expression levels were not affected by either ototoxic, suggesting that the involvement of this enzyme in antioxidation response to both drugs does not require changes in gene expression. Thus, enzymatic antioxidation mechanisms may not be stereotypically affected by aminoglycosides and cisplatin. In this regard, overall diminished antioxidation enzymatic activity has been reported after amikacin ototoxicity in the guinea pig cochlea [40], whereas changes in antioxidation enzymatic activity in the cochlea after cisplatin seem to be more complex, but with low Gpx activity predominating [24]. It is tempting to speculate that diminished cochlear expression of both Gpx1 and Sod1 genes with cisplatin may manifest a larger “exhaustion” of the enzymatic defense systems than with kanamycin.

Cochlear distribution of immunolabeling for the Gpx1 protein product, whose gene expression is down-regulated both by kanamycin and cisplatin, provides additional clues about the involvement of oxidative stress in ototoxicity mediated by these drugs. Although mRNA levels may not bear simple correlations with levels of the corresponding protein [41], patterns of cochlear immunolabeling for Gpx1 support distinct ototoxic oxidative stress responses for kanamycin and cisplatin. With kanamycin, immunolabeling for Gpx1 was diminished in the OHC region throughout treatment, whereas in the stria vascularis, intensity levels at the end of treatment at days 11 and 15 were comparable to controls. In the case of cisplatin, Gpx1 immunolabeling intensity was significantly diminished in the stria vascularis, particularly in the middle and basal turns, at the end of treatment 72 h after the injection, whereas OHCs did not show significant differences with controls. Altogether, this suggests that the GPx1 expression level is downregulated during kanamycin and cisplatin ototoxicity. In the case of kanamycin such down-regulation persists throughout treatment in the OHC region, whereas in the case of cisplatin it seems to more affect the stria vascularis.

## 5. Conclusions

Oxidative stress, including the corresponding enzymatic defense systems, affects different cochlear structures during kanamycin or cisplatin ototoxicity. Oxidative stress in the stria vascularis is of special relevance for cisplatin ototoxicity. These differences may be relevant for the design of targeted treatments. For instance, antioxidant protection of the stria vascularis may be particularly relevant for cisplatin ototoxicity. If the endocochlear potential is preserved and surviving receptor cells in low frequency regions recover their activity, this may be useful to exploit such residual hearing [36].

## Figures and Tables

**Table 1:** List of genes analyzed by RT-qPCR and primer sequences used for transcription amplification.

Gene	Primer	Supplier
*Sod1*	FW: CACTGCAGGACCTCATTTT	
	RV: CACCGTTGCCCAAGTCATCT	Thermo Fisher Scientific
*Gpx1*	FW: GTTTCCCGTGCAATCAGTTC	
	RV: CATTCCGCAGGAAGGTAAAG	Thermo Fisher Scientific
*Cat*	FW: GAGGAAACGCCTGTGTGAGA	
	RV: TTGGCAGCTATGTGAGAGCC	Thermo Fisher Scientific
*Gapdh*	FW: AGACAGCCGCATCATCTTGT	
	RV: CTTGCCGTGGGTAGAGTCAT	Thermo Fisher Scientific

**Table 2:** Primary and secondary antibodies used for immunofluorescence on cochlear cryosections.

Primary Antibodies	Species	Dilution	Supplier (Cat. No.)
3-nitrotyrosine (3-NT)	Mouse	1:100	Abcam (ab61392)
Glutathione Peroxidase 1 (GPX1)	Rabbit	5 µg/mL	Abcam (ab22604)
Secondary antibodies			
Anti-mouse Alexa 488	Donkey	1:200	Thermo-Fisher Scientific (A-21202)
Anti-rabbit Alexa 594	Donkey	1:200	Thermo Fisher Scientific (A-21207)

## Data Availability

The data presented in this study are available in the article.
